# Detection of *Staphylococcus* Isolates and Their Antimicrobial Resistance Profiles and Virulence Genes from Subclinical Mastitis Cattle Milk Using MALDI-TOF MS, PCR and Sequencing in Free State Province, South Africa

**DOI:** 10.3390/ani14010154

**Published:** 2024-01-02

**Authors:** Ntelekwane G. Khasapane, Myburgh Koos, Sebolelo J. Nkhebenyane, Zamantungwa T. H. Khumalo, Tsepo Ramatla, Oriel Thekisoe

**Affiliations:** 1 Centre for Applied Food Safety and Biotechnology, Department of Life Sciences, Central University of Technology, 1 Park Road, Bloemfontein 9300, South Africa; 2 Department of Animal Sciences, Faculty of Natural and Agricultural Sciences, University of the Free State, Bloemfontein 9300, South Africa; myburghj@ufs.ac.za; 3 Vectors and Vector-borne Diseases Research Programme, Department of Veterinary Tropical Diseases, Faculty of Veterinary Science, University of Pretoria, Onderstepoort, Pretoria 0110, South Africa; 4 Unit for Environmental Sciences and Management, North-West University, Potchefstroom 2531, South Africa; 21205450@mynwu.ac.za (T.R.);

**Keywords:** *Staphylococcus*, antimicrobial resistance, virulence, subclinical mastitis, cattle

## Abstract

**Simple Summary:**

Physical injury or microbial infection can cause mastitis, an inflammatory reaction of the udder tissue in the mammary gland of cows. Globally, mastitis is a leading source of significant financial losses for dairy farms. Despite several attempts over the past several years to manage mastitis, efficient and long-lasting control methods or instruments have not yet been created. The current investigation used MALDI-TOF MS and 16S rRNA PCR for the identification of *Staphylococcus* isolates from the milk of cows with subclinical mastitis (SCM), and further screened them for determination of their antimicrobial resistance (AMR) and virulence gene profiles. Our results uncovered that 33.13% of the cows had subclinical mastitis, while the quarter-level prevalence was 54%. Furthermore, MALDI-TOF MS and 16S rRNA PCR assay and sequencing identified *Staphylococcus aureus* as the dominant bacteria. An antimicrobial resistance susceptibility test showed that 86% of the isolates were resistant to penicillin, while antimicrobial resistance and virulence genes showed that the isolates carried mostly the *mecA*- and *Lg G*-binding region genes. The results of this study demonstrate the need for earlier diagnosis and surveillance of SCM and *Staphylococcus* species in the studied area.

**Abstract:**

*Staphylococcus* species are amongst the bacteria that cause bovine mastitis worldwide, whereby they produce a wide range of protein toxins, virulence factors, and antimicrobial-resistant properties which are enhancing the pathogenicity of these organisms. This study aimed to detect *Staphylococcus* spp. from the milk of cattle with subclinical mastitis using MALDI-TOF MS and 16S rRNA PCR as well as screening for antimicrobial resistance (AMR) and virulence genes. Our results uncovered that from 166 sampled cows, only 33.13% had subclinical mastitis after initial screening, while the quarter-level prevalence was 54%. Of the 50 cultured bacterial isolates, MALDI-TOF MS and 16S rRNA PCR assay and sequencing identified *S. aureus* as the dominant bacteria by 76%. Furthermore, an AMR susceptibility test showed that 86% of the isolates were resistant to penicillin, followed by ciprofloxacin (80%) and cefoxitin (52%). Antimicrobial resistance and virulence genes showed that 16% of the isolates carried the *mecA* gene, while 52% of the isolates carried the *Lg G*-binding region gene, followed by *coa* (42%), *spa* (40%), *hla* (38%), and *hlb* (38%), whereas *sea* and *bap* genes were detected in 10% and 2% of the isolates, respectively. The occurrence of virulence factors and antimicrobial resistance profiles highlights the need for appropriate strategies to control the spread of these pathogens.

## 1. Introduction

The Staphylococci that cause mastitis in dairy cattle are divided into two major groups. These are (1) coagulase-positive *Staphylococcus aureus* and (2) Non-aureus staphylococci (NAS). The coagulase-positive *S. aureus* is considered a major pathogenic species whereas NAS are considered minor pathogens [1,2,3]. The majority (About 95%) of coagulase-positive *Staphylococcus* species from bovine mastitis are *S. aureus* [4] but about 15–20% of cases of mastitis are caused by NAS which comprises mainly coagulase-negative (more than 50 species), some coagulase-positive (*S. intermedius, S. psedointermedius, S. coagulans*- they are major pathogens of dogs and cats but also infect humans and occasionally mastitis) and variable [*S. hyicus* (major pathogen of swine [5] but also cause mastitis in dairy cattle), *S. agnetis*- cause mastitis in dairy cattle)] staphylococci [1,6,7,8,9,10]. *Staphylococcus* species, particularly *S. aureus*, are among the most prominent and prevalent etiological agents of bovine mastitis [6,7]. Despite being known as a cause for illnesses with low clinical frequency, non-aureus Staphylococcus (NAS) and Coagulase-negative Staphylococcus have been discovered as frequent pathogens causing mastitis in a number of countries [8,9]. According to Xu et al. [11], it is essential to monitor the epidemiology, prevalence, and incidence of bacteria that cause bovine mastitis, particularly *Staphylococcus* species, to create programs and strategies for protecting human health in accordance with the “One Health” policy and preventing financial loss for dairy producers.

Invasiveness, biofilm formation, toxin-mediated virulence factors, and antibiotic resistance are factors which influence the pathogenicity, cure rate, and ability of these organisms to survive in the host environment [11,12]. Hemolysins, leukocidins, enterotoxins, and superantigens are virulence factors produced by *S. aureus* that promote intramammary infection (IMI) and enable mastitis-causing bacteria to evade the host immune system [13]. Significantly, there are four distinct haemolysin types that *S. aureus* bacteria produce to enhance their pathogenicity, namely, beta, delta, gamma, and toxic shock syndrome toxin-1 (TSST-1), as well as staphylococcal enterotoxins and exfoliative toxins [14,15]. The family *Staphylococcaceae*, which includes the genera *Abyssicoccus*, *Aliicoccus*, *Auricoccus*, *Corticicoccus*, *Jeotgalicoccus*, *Macrococcus*, *Nosocomiicoccus*, *Salinicoccus*, and *Staphylococcus*, has 98 validly documented species as of the writing of this paper, according to Madhaiyan, Wirth, and Saravanan [15]. Gram-positive, non-spore-forming, spherical or coccoid cells with sizes ranging from 0.5 to 2.5 µm are members of this family. They are also non-motile, occurring singly, in pairs, or in tetrads. They are strictly aerobic to facultatively anaerobic, catalase-positive (usually), variable for oxidase, and chemo-organotrophs capable of both fermentative and aerobic metabolism [4]. Staphylococcus, which has 23 subspecies and 55 validly recognised species, is the most common genus in this family [16,17,18,19]. A high frequency of antibiotic resistance (AMR) and a natural reservoir of genes linked to virulence, which particularly favours traits for strains that prove to be more contagious and resistant to antibiotic treatments, are further features shared by all 23 species of coagulase-negative staphylococci (CNS) [20]. Furthermore, CNS are also capable of secreting numerous exotoxins (alpha, beta, gamma, and delta) [21].

Additionally, the existence of antibiotic-resistant bacteria in bovine mastitis cases, as well as the possibility of transmission to humans through consumption of unpasteurized dairy products, are two significant public health concerns. Antibiotics are frequently used to treat mastitis by farmers [22]. However, the overuse of antibiotics in livestock therapy can result in the emergence of antimicrobial-resistant strains and financial losses and further reduce the benefits of mastitis prevention and management [23]. Moreover, *S. aureus*, a bacterium that causes mastitis and is related to food-borne diseases, has also been found to be resistant to several antibiotics [24]. Additionally, the majority of methicillin-resistant *S. aureus* (MRSA) isolates have been detected from humans [25], livestock [26], and the environment [27]. Notably, some bacteria that cause mastitis contain genes that make them resistant to antibiotics, including the *mecA* gene for methicillin resistance [28].

The morphological and biochemical features of the isolates, as well as molecular biology techniques such as PCR combined with Sanger sequencing, which targets the 16S rRNA gene, and comparison of the isolates’ gene sequences with classified references in commonly used databases are generally used to identify bacteria [28]. Due to its advantages over molecular identification methods and biochemical-based tests in terms of speed, cost, and labour savings, MALDI-TOF MS has therefore gained popularity as a substitute method for microbiological identification [28,29,30].

These factors led to the conceptualization of the current investigation, which has used MALDI-TOF MS and 16S rRNA gene sequencing for the identification as well as genetic screening of virulence and antibiotic resistance profiles from *Staphylococcus* isolates obtained from the milk of cows with subclinical mastitis in the Thabo Mofutsanyana District of the Free State Province, South Africa.

## 2. Materials and Methods

### 2.1. Mastitis Screening and Sample Collection

**Figure 1:**
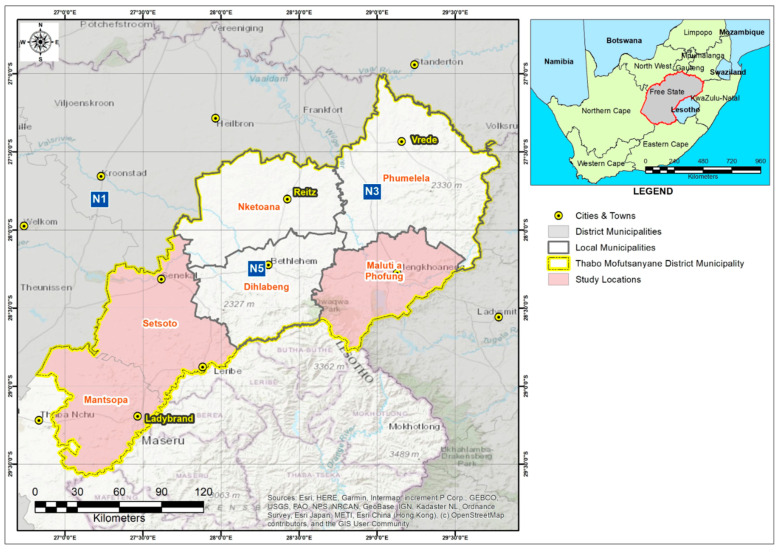
Map showing study area. Pink depicts Mantsopa (latitude: 29.1283° S; longitude: 27.2676° E), Setsoto (latitude: 28.5302° S; longitude: 27.6435° E), and Maluti-a-Phofung (latitude: 28°16′21.94″ S).

Dairy cows from seven small-scale farms spread across three local municipalities, namely, Maluti-a-Phofung, Mantsopa, and Setsoto, were randomly sampled. This resulted in a total of 166 composite milk samples from individual cows, which were screened for intramammary infection using the somatic cell count (SCC) assay via flow cytometry (Mérieux NutriSciences, Midrand, South Africa) (Figure 1). The California Mastitis Test (CMT) (DeLaval, Pinetown, South Africa) was then performed in accordance with the manufacturer’s instructions on only 220 individual quarters from 55 out of 166 cows based on the SCC results from the farm, and subsequently only 160 quarter milk samples were collected in duplicate using sterile 50 mL Falcon tubes., i.e., one batch for another round of the somatic cell count (SCC) assay (Mérieux NutriSciences, South Africa), while another batch was transported tothe laboratory using a cooler box maintained with ice packs for microbiological analysis within 24 h of sampling. Before sample collection, the udder of each cow was washed with distilled water and dried with disposable paper towel to prevent any cross contamination. Thereafter, to ensure that samples were collected ascetically, 70% ethanol was applied on each udder before pure milk samples could be collected [31]. Karzis et al. [32] recommended scoring and interpreting the level of inflammation based on the CMT and SCC results as follows: 0 (negative (healthy quarter), somatic cell count (SCC) ≤ 100,000 cells/mL milk), 1+ (weak positive, SCC > 100,000 < 500,000 cells/mL milk), 2+ (distinct positive, SCC > 500,000 < 1000,000 cells/mL milk), and 3+ (strong positive, SCC ≥ 1000,000 cells/mL milk).

### 2.2. Microbiological Identification of Staphylococcus species

The method of Hoque et al. [33] and Liu et al. [34] was used to identify each bacterial isolate, whereby mannitol salt agar (MSA) plates were streaked with 0.1 mL of mastitic milk samples, and the plates were then incubated at 37 °C for 24 to 48 h. The colonies on MSA were identified based on their morphology (*S. aureus*: yellow colonies with yellow zones; CNS: colourless or red colonies with red zones). Thereafter, we performed Gram staining and a catalase test. Pure cultures were established by subculturing two to three suspected staphylococcal colonies on nutrient agar plates (NAP) and incubating them for 24 to 48 h at 37 °C. In addition, while further experiments were conducted, all pure *Staphylococcus* isolate colonies were maintained at −80 °C in BHI broth with 15% glycerol.

### 2.3. Detection of Staphylococcus Species Using MALDI-TOF Method

Staphylococcal species or genera were identified using the Biotyper 3.1 program (Bruker, Johannesburg 2191, South Africa). The *Escherichia coli* DH5α Bacterial Test Standard (BTS) was used to calibrate the Autoflex Speed equipment (Bruker Daltonics, Billerica, MA, USA). Additionally, all bacterial isolates were identified according to Cameron et al. [35,36]. Analysis for each isolate was run in duplicate. Isolates were declared unidentified if they were not resolved after 2 rounds of MALDI-TOF MS analysis. For reliability of our analysis, a cut-off score ≥ 1.7 was used as a threshold for the bacterial identification [35,36].

### 2.4. DNA Extraction and PCR

In line with the manufacturer’s instructions, genomic DNA from the appropriate isolates was extracted for this investigation using the Quick-DNA^TM^ Fungal/Bacterial Miniprep Kit (Zymo Research, Tustin, CA 92614, USA). The concentration of DNA was measured using a NanoDrop^TM^ 2000 Spectrophotometer (Oxoid, ThermoFisher, Johannesburg, South Africa). All PCR studies employed two controls, that is, *S. aureus* ATCC 25923 (positive control) and nuclease-free water (negative control).

### 2.5. Molecular Identification of Isolates

Primers 27F 5′-AGAGTTTGATCCTGGCTCAG-3′ and 1492R 5′-TACCTTGTTACGACTT-3′ [37,38,39] were used for the identification of *Staphylococcus* spp. using 16S rDNA PCR assay. Thermonuclease (*nuc*) and elongation factor Tu (*tuf*) genes were utilized as species-specific genes for both *S. aureus* and CNS, respectively, using published primers (Table 1). Amplification was conducted in a 12.5 μL reaction volume consisting of 4.25 μL of OneTaq 2× master mix with standard buffer (New England Biolabs, Ipswich, MA, USA), 2 μL of DNA template (1–2 ng), 1 μL each forward and reverse primers, and 4.25 μL of double-distilled water using a ProFlex PCR System (Applied Biosystems, Framingham, MA, USA).

PCR conditions were in accordance with the published work of Liu et al. [34], with a few modifications. The PCR amplicons were observed under ultraviolet (UV) light on a 1% agarose gel stained with ez-vision^®^ (Bronx, NY, USA) bluelight DNA dye. The 1 Kb and 100 bp DNA ladders (Sigma, Shanghai, China, D7058) were used as molecular markers. The16S rRNA PCR amplicons were submitted at Inqaba Biotechnical Industries (Pty) Ltd., Pretoria, South Africa, for Sanger sequencing [30].

### 2.6. Detection of mecA Gene from Staphylococcus Isolates

All isolates were further screened for the *mecA* gene encoding for methicillin antibiotic resistance traits. Primers were as follows: mecA-1: 5′-AAAATCGATGGTAAAGGTTGGC-3′; mecA-2: 5′-AGTTCT GCAGTACCGGATTTGC3′. The amplicon sizes were 533 bp [40]. PCR was performed in a 12.5 μL reaction volume containing OneTaq 2× master mix with standard buffer (New England Biolabs, Ipswich, MA, USA) (4.25 μL), genomic DNA (1–2 ng) (2 μL), 1 μL each of 10 μM forward primer and 10 μM reverse primer, and nuclease-free water (4.25 μL) with the following thermocycling conditions: 94 °C for 2 min, followed by 25 cycles of 94 °C for 15 s, 55 °C for 30 s, 72 °C for 30 s, and 72 °C for 10 min [40] using ProFlex PCR System (Applied Biosystems, Waltham, MA, USA).

### 2.7. Detection of Virulence Genes

For the purpose of this study, all isolates were further screened for virulence genes, namely, *lg*, *spa*, *coa*, *bap*, *hla*, *hlb*, and *sea*, using published primers (Table 1). The binding of immunoglobulin G (*Lg G*) is known for its involvement in the neutralization and elimination of microbes. The *Lg G*-binding polyptite gene is associated with the Lg G-binding protein, while the *spa* gene encodes for staphylococcal protein-A. Lg G-binding ability is common among clinical strains of *S. aureus*. Lastly, *bap* and *coa* genes encode for biofilm-associated protein and coagulase, respectively, while *hla*, *hlb*, and *sea* encode for haemolysins and enterotoxins.

Individual PCR assays for each molecular identification of virulent and antimicrobial genes were set up in a 12.5 μL volume. The PCR reaction and conditions are described above in Section 2.5.

### 2.8. Antimicrobial Susceptibility Testing

Disk diffusion was performed for isolate phenotypic susceptibility testing using only four antibiotics on a 90 mm plate [37]. After being subcultured on nutritional agar (Merck, Wadeville, South Africa), pure *Staphylococcus* isolates were then incubated for 24 h at 37 °C. Thereafter, tests for antibiotic sensitivity were conducted using newly propagated overnight cultures. Using a sterile cotton swab, 100 μL aliquots from the suspensions were spread-plated on Mueller Hinton agar (MH), and the plates were incubated at 37 °C for 24 h. To evaluate the susceptibility of *Staphylococcus* isolates to widely used antimicrobial drugs, the single disk diffusion technique was utilized. Antibiotic discs (ThermoFisher, South Africa) comprising gentamicin (CA, 10 μg), ampicillin (AMP, 10 μg), tetracycline (TE, 30 μg), penicillin (P, 10 μg), erythromycin (E, 15 μg), ciprofloxacin (CIP, 5 μg), and cefoxitin (FOX, 15 μg) were utilized in this investigation. The Clinical Laboratory Standards Institute [42] guidelines, which are interpreted as intermediate (I), sensitive (S), and resistant (R) (Table 2), were used to assess the antimicrobial profile of isolated staphylococci to different antibiotics using the quality control strain *Staphylococcus aureus* ATCC 25923 [16]. Multidrug-resistant (MDR) isolates were defined as those that exhibited resistance to at least three different classes of antibiotics [37].

### 2.9. Data Analysis

The antimicrobial resistance of an isolate was calculated as the percentage of isolates among the group that were resistant to a single antibiotic or a number of antibiotics [22]. We further used tables and graphs to display the relationships of different variables and also for comparative analysis of data on antimicrobial resistance pattern of *Staphylococcus* isolates. The heatmap plots of the antibiotic resistance profile were generated using ChipPlot (https://www.chiplot.online/#, accessed on 30 October 2023).

## 3. Results

### 3.1. California Mastitis Test (CMT) and Somatic Cell Count (SCC)

Out of the 166 cows that were sampled, only 55 (33.13%) were positive for subclinical mastitis at cow level based on CMT. Moreover, only 87 (54%) quarter-level samples were positive for subclinical mastitis based on the SCC assay.

### 3.2. Bacterial Isolation and Identification

Based on phenotypic characteristics, the catalase test, and the mannitol salt agar tests, 70 presumptive isolates were suspected to be *Staphylococcus* species. Thereafter, 50 selected isolates were identified using MALDI-TOF MS according to Cameron et al. [35] using the Biotyper algorithm (Bruker Daltonics, Bremen, Germany). Our results showed that *S. aureus* was the dominant species with 38/50 (76%) isolates, followed by *Staphylococcus chromogenes* 6/50 (12%), *Staphylococcus epidermidis* 2/50 (4%), and *Staphylococcus haemolyticus* 2/50 (4%); thereafter, we had mixed cultures between *S. aureus* and *Staphylococcus hyicus* 2/50 (4%) (Table 3).

We further continued to confirm *Staphylococcus* isolates using 16S rRNA gene and species-specific *tuf* gene for coagulase-negative staphylococci and *nuc* gene for *S. aureus* by means of PCR. Both PCR assays and amplicon sequences revealed that isolates of the current study were *S. aureus*, *S. chromogenes*, *Staphylococcus agnetis*, *Staphylococcus argenteus*, and *Staphylococcus devriesei* at 38/50 (76%), 5/50 (10%), 4/50 (4%), 2/50 (4%), and 1/50 (1%), respectively (accession numbers provided in Supplementary Table S1).

### 3.3. Detection of Methicillin Resistance and Virulence Genes

**Figure 2:**
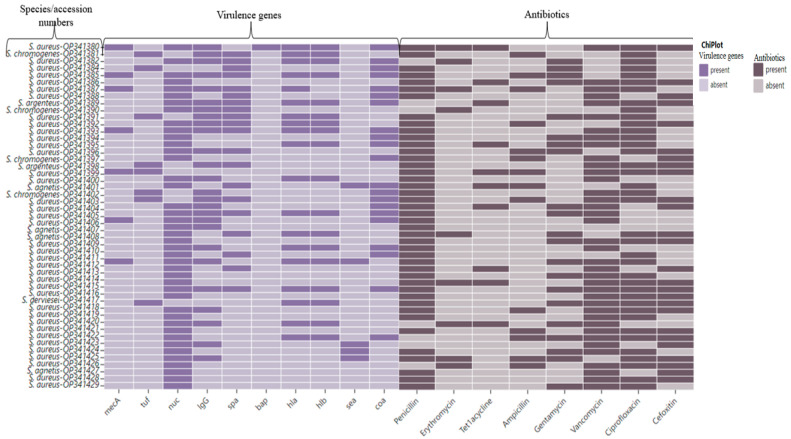
Heatmap showing the clustering of the antibiotic resistance and virulence profiles in the *Staphylococcus* species isolates. The blue colour indicated the presence of virulence genes (*Lg G*, *spa*, *coa*, *bap*, *hla*, *hlb*, and *sea*) and antibiotic resistance gene (*mecA*). Brown colour indicates the presence of antibiotic resistance.

Out of 50 genomic DNA samples that were randomly selected, the *mecA* gene encoding for methicillin resistance was amplified in 8/50 (16%) isolates. Thereafter, we screened 50 selected isolates for virulence genes using PCR. The results of this investigation revealed that 52% (26/50) of all the isolates carried the Lg G-binding region gene, followed by 42% (21/50) and 40% (20/50) of the isolates carrying the *coa* and *spa* genes, respectively. The *hla* and *hlb* genes were both detected in 38% (19/50) of the isolates, whereas the *sea* and *bap* genes were detected in 10% (5/50) and 2% (1/50) of the isolates, respectively. These values can be seen in Table 4 and Figure 2.

### 3.4. Phenotypic Antimicrobial Resistance Test

The distribution of the antibiotic resistance of each *Staphylococcus* isolate is shown in the heatmap (Figure 2). The 43/50 (86%) isolates showed resistance to penicillin, 40/50 (80%) to ciprofloxacin, 39/50 (76%) to vancomycin, and 26/50 (52%) to cefoxitin. Resistance against gentamycin, ampicillin, tetracycline, and erythromycin was observed in 18/50 (36%), 14/50 (28%), 9/50 (18%), and 9/50 (18%), respectively.

Among all staphylococcal species, *S. aureus* had the highest percentage of resistance to all antibiotics followed by *S. chromogenes*, *S. agnetis*, *S. argenteus*, and *S. devriesei* (Table 5).

Multidrug resistance analysis showed that *S. aureus* had the highest number of resistant isolates at 34, followed by *S. chromogenes* with 5 isolates. Lastly, *S. agnetis* had four isolates with MDR, followed *S. argenteus* and *S. derviesei* with one isolate each as depicted in Table 6.

## 4. Discussion

Since *S. aureus* and CNS may both be detected in raw milk without raising SCC and are also found on the udders of cows, they are commonly linked to intramammary infection (IMI) [44]. Our CMT (33.13%) and SCC (54%) results were relatively lower than those previously documented in other parts of Africa, i.e., Uganda, Kenya, and Ethiopia, at 86.2%, 64%, and 59.2%, respectively [38,39,40]. Further analysis showed that every sample collected had *Staphylococcus* spp. after microbiological culturing. MALDI-TOF MS and 16S rRNA gene sequencing revealed that *S. aureus* and other CNS are species occurring in the current study’s samples. This investigation supports the claims of Braga et al. [45] that MALDI-TOF MS is a better method compared to traditional biochemical testing for classifying *Staphylococcus* isolates. Nevertheless, the same author [46] further narrated that some staphylococci species, such as *M. Sciuri*, *S. xylosus*, and *S. equorum*, cannot be identified at the species level using MALDI-TOF MS but can instead be identified using other methods, such as DNA-based techniques. This was in line with multiple studies that reported on this restriction of MALDI-TOF MS technology [47,48]. Furthermore, *S. aureus* was the dominant species, followed by *S. chromogenes*, *S. agnetis*, *S. argenteus*, and *S. devriesei*, according to the current study’s 16S rRNA Sanger sequencing data; this method has been used in multiple studies to identify *S. aureus* isolates from various sources [49,50]. When the gene encoding a 16S rRNA gene was amplified, all of the *S. aureus* isolates under investigation had an amplicon size of more than 1250 bp, which validates findings from other studies [51,52].

In the current study, the two analytical tools (MALDI-TOF MS and 16S rRNA sequencing) produced similar results for most identified organisms; however, some isolates were identified as mixed cultures by MALDI-TOF MS. This is due to the fact that not all *Staphylococcus* isolates were tested for coagulase and *S. aureus* can be coagulase-negative, amongst other reasons [16,41]. Additionally, it has been demonstrated that the MALDI-TOF MS identification is influenced by variables including cell wall rigidity, growth phase, and culture conditions, including selective media that may have impact on the observed protein expression and cell concentration [42], where a cut-off below 2.0 enhances misidentification of envisaged pathogens [43]. These are some of the limitations incurred by biochemical techniques. For all comparison tests, we utilized one pure colony for both MALDI-TOF MS analysis and the 16S rRNA sequencing. This was an effort to rule out the idea that several microorganisms could be separated, which would appear to cause test disagreement. The current study might have had problems with the detection of nonviable bacteria; however, MALDI-TOF MS and 16S rRNA sequencing methods can also detect nonviable organisms, which can be problematic when trying to diagnose active cases of mastitis [44,46].

*Staphylococcus aureus* is a well-known bacterium that poses as a hazard to both human and animal health by generating potentially serious infections. More pathogenic strains of *S. aureus* are thought to be able to use the antibiotic resistance genes that may be present in CNS [53,54,55,56,57,58,59,60,61,62,63,64]. The isolates have shown resistance to penicillin 43/50 (86%), followed by ciprofloxacin 40/50 (80%), and cefoxitin 26/50 (52%). The observed resistance against gentamicin, ampicillin, tetracycline, and erythromycin was 18/50 (36%), 14/50 (28%), 9/50 (18%), and 9/50 (18%), respectively. The results of the current study are somewhat similar to those of Sundareshan et al. [65], who reported that there were more staphylococci with penicillin resistance in subclinical mastitis (63%) in dairy cows of India. Our findings, however, did not support those of Schmidt et al. [66], who discovered that 48% of *S. aureus* isolates in the KwaZulu-Natal province of South Africa were beta-lactam-resistant. Moreover, due to its use in treating MRSA cases, our analysis found that gentamicin resistance also occurred in the isolates, which was consistent with a study by Martins et al. [67] that found *S. aureus* to have 12.50% resistance to gentamicin while CNS had just 3.45% resistance in Brazil. Additional research on AMR in CNS is required due to the potential emergence of new resistance mechanisms, which poses a problem for the management of bovine mastitis cases. The acquisition of the staphylococcal cassette chromosome *mec*, a mobile genetic element, contributed to the development of methicillin resistance in *S. aureus*. According to Turner et al. [68], this cassette contains the *mecA* gene, which controls the development of low-affinity penicillin-binding protein 2a (PBP2a) and gives pathogens resistance to beta-lactamase antibiotics. In our investigation, *mecA* was found in 16% of *S. aureus* isolates linked to mastitis in cattle. The *mecA* gene was detected in 35.70% and 74.08% of *S. aureus* isolates in China and India, respectively, according to Xu et al. [10] and Patel et al. [69]. Furthermore, Castro et al. [70] and Monistero et al. [71] all reported lower levels of the *mecA* gene compared to other AMR genes in Brazil and South Africa, respectively.

To ascertain the pathogenicity of the species, we also checked for the occurrence of virulence genes. The gangrenous type of mastitis, which involves a restriction in blood flow to mammary tissues and subsequent injury to smooth muscles, is known to be brought on by *hla* expression, which is linked to the toxin a-haemolysin [70]. The *hlb* gene is associated with the neutral sphingomyelinase toxin b-haemolysin, which has been shown by Singh et al. [72] and Neelam et al. [22] to be responsible for the breakdown of sphingomyelin in the cell membranes of erythrocytes, leukocytes, neurons, and other tissue cells. B-haemolysin promotes the development of biofilms [66], increases *S. aureus* adhesion to the epithelium of the bovine mammary gland, and increases resistance to antimicrobials [71]. The presence of *hla* and *hlb* genes at 38% in *S*. *aureus* isolates in the present study are in close agreement with previous findings in Egypt (34.4% and 43.75%) [72], Brazil (38% and 58%) [73], and China (57% and 36%) [66]. However, they are comparatively lower than several studies conducted in China, where the *hla* and *hlb* genes were both detected in over 80% of the isolates [74,75,76,77].

Enterotoxin production contributes to the pathophysiology of many human diseases, including toxic shock syndrome, pneumonia, sepsis, and food poisoning epidemics; hence, *S. aureus* strains are also regarded as major foodborne pathogens. Although it is unclear how enterotoxins contribute to the aetiology of bovine mastitis, their presence in milk can be a severe public health problem. Even after milk has been pasteurized, enterotoxins maintain their biological activity because they are stable at high temperatures [15]. Zschock et al. [78] further alluded to those enterotoxins, which can cause diarrhoea and other difficulties in humans and are produced by enterotoxigenic staphylococci-infected animals’ udders, which are then consumed as milk. More than 90% of food poisoning outbreaks linked to *S. aureus* were associated with traditional staphylococcal enterotoxins [75,76]. The current study investigated the occurrence of the staphylococcal enterotoxin *sea* gene in each *S. aureus* isolate, and it was detected in 10% of the isolates. Our observations are in agreement with those of previous studies, which reported the *sea* gene in 7.10%, 10.9%, and 19.4% of samples in China, Brazil, and Czech Republic; however, these were much lower than those detected in South Africa (35.29%), northern Egypt (52%), and Italy (65.60%) from raw meats in retail markets [10,77,78].

*Staphylococcus aureus* attaches to the surface of the host cell to begin the colonization process via adhesins that the bacteria have on their surface [79]. The majority of the adhesins present in *S. aureus* are protein A proteins in the X-region and Lg G-binding regions that are found in cell peptidoglycan (*spa*) [80]. Due to its ability to attach to molecules and agglutinate bacteria against particular bacterial antigens, protein A is utilized as a crucial reagent in immunology and diagnostic laboratory technology because it can attach to molecules and agglutinate bacteria against a particular antigen [81]. The *spa* gene produces protein A as a result of this process. The investigation indicated that 20 isolates were positive for the presence of the *spa* (X-region) gene. The isolates containing the *spa* gene were found to form bands of various widths such as 130 bp, 31 of 200 bp, 16 of 290 bp, and 13 of 310 bp. As a result, it was discovered that 40% of the isolates possess the *spa* gene; however, there are gene variations. Furthermore, these genes have been detected in numerous studies focusing on bovine mastitis, which may entail the frequent presence of these genes in *S. aureus* [82].

In the current study, *S. aureus* was the predominant bacterium identified followed by *S. chromogenes*, *S. argenteus*, *S. agnetis*, *S. haemolyticus*, and *S. devriesei* from subclinical mastitis cows using both MALDI-TOF MS- and PCR-based techniques. This study further showed that most of the isolates carried the *Lg G*-binding protein gene, followed by *coa* and *spa* (X-region), reiterating their public health importance. A total of 45 bacterial isolates showed a trend in acquiring MDR such as penicillin, ciprofloxacin, vancomycin, and cefoxitin. Hence, the notion that we had 26 isolates that were phenotypically resistant to cefoxitin led us to investigate whether these isolates are indeed genetically resistant to methicillin; thus, we found 8 isolates that had the *mecA* gene. As limitations, the current study did not analyse phenotypic expressions of the virulence factors and subclinical mastitis risk factors such as breed, parity, age, farm system, or other farm characteristics. Furthermore, this study was only limited to the analysis of only 50 randomly selected isolates due to financial constraints.

## 5. Conclusions

The data generated in this study highlight the necessity for improved early detection and surveillance of *Staphylococcus* isolates and subclinical mastitis. Furthermore, this study showed the need for collaboration between stakeholder such as the dairy farming community, veterinary and human health sectors, as well as environmental scientists for improved control and prevention.

## Figures and Tables

**Table 1:** Primer sequences (F, forward; R, reverse) specific to different putative virulence and antimicrobial resistance genes in staphylococcal isolates.

Target Gene	Primer Sequences (5′-3′)	Product (bp)	Reference
*tuf* (Elongation factor Tu)	F-CCAATGCCACAAACTCGT	412 bp	[40]
	R-CCTGAACCAACAGTACGT		
*Nuc* (Thermonuclease)	F-CGATTGATGGTGATACGGTT	279 bp	[41]
	R-ACGCAAGCCTTGACGAACTAAAGC		
*spa* (Lg G-binding region)	F-CACCTGCTGCAAATGCTGCG	920 bp	[41]
	R-GGCTTGTTGTTGTCTTCCTC		
*spa* (X-region)	F-CAAGCACCAAAAGAGGAA	Variable (220, 253, 315 bp)	[42]
	R-CACCAGGTTTAACGACAT		
*coa* (Coagulase)	F-ATAGAGATGCTGGTACAGG	Variable (627, 710, 910 bp)	[42]
	R-GCTTCCGATTGTTCGATGC		
*bap* (Biofilm-associated protein)	F-CCCTATATCGAAGGTGTAGAATTG	971 bp	[42]
	R-GCTGTTGAAGTTAATACTGTACCTGC		
*hla* (Hemolysin A)	F-GGTTTAGCCTGGCCTTC	550 bp	[43]
	R-CATCACGAACTCGTTCG		
*hlb* (Hemolysin B)	F-GCCAAAGCCGAATCTAAG	840 bp	[43]
	R-GCGATATACATCCCATGGC		
*sea* (Enterotoxin A)	F-GCAGGGAACAGCTTTAGGC	521 bp	[43]
	R-GTTCTGTAGAAGTATGAAACACG		

**Table 2:** Zone diameter breakpoints for *Staphylococcus* isolate.

Antibiotic Disks	Concentration (µg)	Abbreviations	Resistant (mm)	Intermediate (mm)	Susceptibility (mm)
Ampicillin	10	AMP	≤28	……	≥29
Ciprofloxacin	5	CIP	≤15	16–20	≥21
			≤15	16–18	≥19
			≤20	21–23	≥24
Erythromycin	15	E	≤13	14–22	≥23
			≤13	14–17	≥18
			≤13	14–17	≥18
Gentamicin	10	CN	≤12	13–14	≥15
Penicillin	10	P	≤28	……	≥29
Tetracycline	30	TET	≤14	15–18	≥19
Cefoxitin	30	FOX	≤24	……	≥25

**Table 3:** Bacterial isolates from subclinical bovine mastitis identified via MALDI-TOF MS.

Bacteria	Log Score ^1^	Bacteria	Log Score ^1^	Bacteria	Log Score ^1^	Bacteria	Log Store ^1^	Bacteria	Log Score
*S. aureus*	≥2	*S. chromogenes*	≤2	*S. chromogenes*	≥2	*S. aureus*	≥2 ≤2	*S. aureus*	≤2
*S. aureus*	≥2	*S. chromogenes*	≤2	*S. aureus*	≤2	*S. aureus*	≤2 ≥2	*S. aureus*	≤2
*S. aureus*	≥2	*S. aureus*	≥2	*E. faecalis*	≥2 ≤2	*S. aureus*	≤2 ≥2	*S. aureus/S. hyicus*	≤2
*S. aureus*	≥2	*S. aureus*	≤2	*S. aureus*	≥2	*S. aureus*	≥2	*S aureus*	≥2
*S. aureus*	≤2	*S. aureus*	≤2	*S. aureus*	≥2	*S. haemolyticus*	≥2	*S. aureus/S. hyicus*	≤2
*S. aureus*	≥2	*S. aureus*	≤2	*S. aureus*	≥2	*S. haemolyticus*	≤2	*S. aureus*	≥2
*S. aureus*	≥2	*S. chromogenes*	≤2	*S. aureus*	≥2	*S. chromogenes*	≤2	*S. aureus*	≤2
*S. aureus*	≥2	*S. aureus*	≤2	*S. aureus*	≥2	*S. aureus*	≥2	*S. aureus*	≥2
*S. aureus*	≤2	*S. epidermidis*	≤2	*S. aureus*	≤2	*S. chromogenes*	≤2	*C. oceanisediminis*	≤2
*S. aureus*	≥2	*S. epidermidis*	≤2	*S. aureus*	≥2	*S. aureus*	≥2	*S. aureus*	≥2

^1^ A log score between 0 and 3 is calculated with the Biotyper algorithm (Bruker Daltonics, Bremen, Germany). Log scores < 1.7 provide no identification. Log score ≥ 1.7 x < 2.0 indicates genus identification, and log score ≥ 2 indicates species identification. All identifications ≥ 2.0 expressed specific species identification. Next-closest score always specifies the same species if the score was ≥2.0.

**Table 4:** Percentage of detected virulence genes from *Staphylococcus* species.

Virulence Genes	Species				
	*S. aureus*	*S. chromogenes*	*S. agnetis*	*S. argenteus*	*S. devriesei*
*Lg G*	20 (40%)	4 (8%)	1 (2%)	1 (2%)	-
*spa*	13 (26%)	5 (10%)	1 (2%)	1 (2%)	
*coa*	18 (36%)	2 (4%)	1 (2%)	-	-
*bap*	1 (2%)	-	-	-	-
*hla*	15 (30%)	2 (4%)	1 (2%)	-	1 (2%)
*hlb*	15 (30%)	2 (4%)	1 (2%)	-	1 (2%)
*sea*	5 (10%)	-	-	-	-

**Table 5:** Percentage of AMR among *Staphylococcus* species.

Antibiotics	Species				
	*S. aureus* (*n* = 38)	*S. chromogenes* (*n* = 5)	*S. agnetis* (*n* = 4)	*S. argenteus* (*n* = 2)	*S. devriesei* (*n* = 1)
Penicillin	32 (84.2%)	5 (100%)	4 (100%)	1 (50%)	1 (100%)
Erythromycin	6 (15.7%)	−	1 (25%)	1 (50%)	−
Tetracycline	9 (23.68%)	−	−	1 (50%)	−
Ampicillin	10 (26.31%)	2 (40%)	−	1	1 (100%)
Gentamycin	15 (39.47%)	2 (40%)	1 (25%)	−	−
Ciprofloxacin	30 (78.94%)	5 (100%)	4 (100%)	2 (100%)	1 (100%)
Cefoxitin	19 (50%)	2 (40%)	3 (75%)	1 (50%)	1 (100%)

**Table 6:** *Staphylococcus* AMR and the prevalence of resistant strains.

AMR Phenotypes	Isolates				
	*S. aureus*	*S. chromogenes*	*S. agnetis*	*S. argenteus*	*S. devriesei*
P, CIP	3	−	1	−	−
P, FOX	1	−	1	−	−
P, CN,	1	−	−	−	−
P, CIP, CN	−	1	−	−	−
P, FOX, AMP	1	−	−	−	−
P, CIP, FOX	−	1	−	−	−
P, CIP, AMP	1	1	−	−	−
P, CIP, CN	1	1	−	−	−
P, CIP, AMP	1	−	−	−	−
P, CIP, TET	1	−	−	−	−
P, FOX, E	−	−	1	−	−
P, CIP, CN	1	−	−	−	−
P, FOX, TE,	1	−	−	−	−
P, CIP, CN	1	−	−	−	−
P, CIP, FOX	1	−	−	−	−
CN, CIP, E	1	−	−	−	−
P, CIP, CN, AMP	1	−	−	−	−
P, CIP, AMP, TET	1	−	−	−	−
TET, CIP, FOX	2	−	−	−	−
P, CIP, AMP, E	1	−	−	−	−
P, CIP, GEN, TET	1	−	−	−	−
P, CIP, FOX, CN, AMP	1	−	−	−	−
P, CIP FOX, AMP	1	1	−	−	−
P, CIP, FOX, CN	−	−	1	−	−
P, CIP, FOX, GEN	1	−	−	−	−
P, CIP, FOX, AMP	1	−	−	−	1
CIP, FOX, TET, E	1	−	−	−	−
CIP, FOX, CN, AMP	1	−	−	−	−
P, CIP, FOX, CN, E	1	−	−	−	−
CIP, FOX, AMP, E	1	−	−	−	−
P, CIP, FOX, TET, E	1	−	−	−	−
P, CIP, FOX, CN, TET	1	−	−	−	−
P, CIP, FOX, AMP, TET	1	−	−	1	−
P, CIP, FOX, TET, E	1	−	−	−	−

## Data Availability

The data used to support the findings of this study are available in the present manuscript.

## Supplementary Materials

The following supporting information can be downloaded at https://www.mdpi.com/article/10.3390/ani14010154/s1: Table S1: Accession numbers of *Staphylococcus* isolates.
