# Supplementary material for: Detection of Staphylococcus Isolates and Their Antimicrobial Resistance Profiles and Virulence Genes from Subclinical Mastitis Cattle Milk Using MALDI-TOF MS, PCR and Sequencing in Free State Province, South Africa
Source: Animals (Basel). 2024 Jan 2;14(1):154. doi: 10.3390/ani14010154 (PMC10778211; doi:10.3390/ani14010154)
Supplement: Supplementary file 1 [file animals-14-00154-s001.zip › animals-2720469-supplementary.pdf]

Isolates ID	Sequence length	16S rRNA results	Accession number
1.1	1450	*S. aureus*	OP341380
1.2	1453	*S. chromogenes*	OP341381
1.3	1464	*S. aureus*	OP341382
1.4	1451	*S. chromogenes*	OP341383
1.5	1443	*S. aureus*	OP341384
1.6	1453	*S. aureus*	OP341385
1.7	1456	*S. aureus*	OP341386
1.8	1454	*S. aureus*	OP341387
1.9	1452	*S. aureus*	OP341388
1.10	1455	*S. argenteus*	OP341389
2.1	1455	*S. chromogenes*	OP341390
2.2	1430	*S. aureus*	OP341391
2.3	1438	*S. aureus*	OP341392
2.4	1454	*S. aureus*	OP341392
2.5	1456	*S. aureus*	OP341393
2.6	1454	*S. aureus*	OP341394
2.7	1437	*S. aureus*	OP341395
2.8	1435	*S. chromogenes*	OP341396
2.9	1455	*S. argenteus*	OP341397
2.10	1445	*S. aureus*	OP341398
3.1	1461	*S. aureus*	OP341399
3.2	1329	*S. agnetis*	OP341400
3.3	1445	*S. chromogenes*	OP341401
3.4	1448	*S. aureus*	OP341402
3.5	1418	*S. aureus*	OP341403
3.6	1461	*S. aureus*	OP341404
3.7	1459	*S. aureus*	OP341405
3.8	1454	*S. agnetis*	OP341406
3.9	1450	*S. agnetis*	OP341407
3.10	1451	*S. aureus*	OP341408
4.1	1441	*S. aureus*	OP341409
4.2	1450	*S. aureus*	OP341410
4.3	1434	*S. aureus*	OP341411
4.4	1458	*S. aureus*	OP341412
4.5	1423	*S. aureus*	OP341412
4.6	1458	*S. aureus*	OP341414
4.7	1451	*S. aureus*	OP341415
4.8	1455	*S. derviesei*	OP341416
4.9	1439	*S. aureus*	OP341417
4.10	1425	*S. aureus*	OP341418
5.1	1451	*S. aureus*	OP341419
5.2	1451	*S. aureus*	OP341420
5.3	1423	*S. aureus*	OP341421
5.4	1455	*S. aureus*	OP341422
5.5	1450	*S. aureus*	OP341423
5.6	1456	*S. aureus*	OP341424
5.7	1430	*S. aureus*	OP341425
5.8	871	*S. agnetis*	OP341426
5.9	1448	*S. aureus*	OP341427

5.10	1454	*S. aureus*	OP341389
